# Clinical and biochemical endpoints and predictors of response to plasma exchange in septic shock: results from a randomized controlled trial

**DOI:** 10.1186/s13054-022-04003-2

**Published:** 2022-05-12

**Authors:** Klaus Stahl, Philipp Wand, Benjamin Seeliger, Pedro David Wendel-Garcia, Julius J. Schmidt, Bernhard M. W. Schmidt, Andrea Sauer, Felix Lehmann, Ulrich Budde, Markus Busch, Olaf Wiesner, Tobias Welte, Hermann Haller, Heiner Wedemeyer, Christian Putensen, Marius M. Hoeper, Christian Bode, Sascha David

**Affiliations:** 1 Department of Gastroenterology, Hepatology and Endocrinology, Hannover Medical School, Hannover, Germany; 2 Department of Nephrology and Hypertension, Hannover Medical School, Hannover, Germany; 3 Department of Respiratory Medicine and German Centre of Lung Research (DZL), Hannover Medical School, Hannover, Germany; 4 Institute of Intensive Care Medicine, University Hospital Zurich, Zurich, Switzerland; 5 Department of Anesthesiology and Intensive Care Medicine, University Hospital Bonn, Bonn, Germany; 6 Medilys Laborgesellschaft mbH, Hamburg, Germany

**Keywords:** Extracorporeal treatment[, Plasmapheresis, Endothelium, Blood purification, Fresh frozen plasma, Sepsis, Precision medicine, Personalized medicine

## Abstract

**Background:**

Recently, a randomized controlled trial (RCT) demonstrated rapid but individually variable hemodynamic improvement with therapeutic plasma exchange (TPE) in patients with septic shock. Prediction of clinical efficacy in specific sepsis treatments is fundamental for individualized sepsis therapy.

**Methods:**

In the original RCT, patients with septic shock of < 24 h duration and norepinephrine (NE) requirement ≥ 0.4 μg/kg/min received standard of care (SOC) or SOC + one single TPE. Here, we report all clinical and biological endpoints of this study. Multivariate mixed-effects modeling of NE reduction was performed to investigate characteristics that could be associated with clinical response to TPE.

**Results:**

A continuous effect of TPE on the reduction in NE doses over the initial 24 h was observed (SOC group: estimated NE dose reduction of 0.005 µg/kg/min per hour; TPE group: 0.018 µg/kg/min per hour, *p* = 0.004). Similarly, under TPE, serum lactate levels, continuously decreased over the initial 24 h in the TPE group, whereas lactate levels increased under SOC (*p* = 0.001). A reduction in biomarkers and disease mediators (such as PCT (*p* = 0.037), vWF:Ag (*p* < 0.001), Angpt-2 (*p* = 0.009), sTie-2 (*p* = 0.005)) along with a repletion of exhausted protective factors (such as AT-III (*p* = 0.026), Protein C (*p* = 0.012), ADAMTS-13 (*p* = 0.008)) could be observed in the TPE but not in the SOC group. In a multivariate mixed effects model, increasing baseline lactate levels led to greater NE dose reduction effects with TPE as opposed to SOC (*p* = 0.004).

**Conclusions:**

Adjunctive TPE is associated with the removal of injurious mediators and repletion of consumed protective factors altogether leading to preserved hemodynamic stabilization in refractory septic shock. We identified that baseline lactate concentration as a potential response predictor might guide future designing of large RCTs that will further evaluate TPE with regard to hard endpoints.

*Trial registration* Retrospectively registered 18th January 2020 at clinicaltrials.gov (Identifier: NCT04231994).

**Supplementary Information:**

The online version contains supplementary material available at 10.1186/s13054-022-04003-2.

## Background

Sepsis is defined as life-threatening organ dysfunction caused by a dysregulated host response to infection and if hypotension is refractory to volume resuscitation with concurrent elevation of serum lactate it is termed septic shock [1]. In the absence of a specific intervention other than anti-infectives, mortality remains exceedingly high [2]. Although the overwhelming host response has been recognized as a key underlying pathophysiological concept in sepsis [3], there still exists no specific treatment option for this causative target [4]. Part of the failure to develop effective specific therapeutic strategies might be attributable to the complexity and nonlinearity of sepsis pathophysiology making it unlikely for a single agent to successfully influence and rebalance the host response [5].

The theoretical concept of adjunctive therapeutic plasma exchange (TPE) in sepsis combines two major aspects in a singular intervention [6, 7]: First, the removal of injurious circulating molecules that directly contribute to the manifestation of the disease, including pro-inflammatory (Interleukin (IL)-6), permeability inducing (e.g., Angiopoietin-2) and pro-coagulative (e.g., Willebrand factor (vWF) antigen) factors [8, 9] and second, but equally important, the replacement of protective plasma proteins that compensate for the sepsis-associated loss of factors important for coagulation (e.g., activated protein C, antithrombin), fibrinolysis (e.g., vWF cleaving proteases) and counteract inflammation and vascular leakage (e.g., Angiopoietin-1, immunoglobulins) [8–10]. A meta-analysis identified four single-center randomized controlled trials (RCTs) that analyzed TPE in sepsis and found that TPE was associated with a reduced mortality in adult patients [11]. However, the largest of those trials, which showed a trend toward improved survival, was underpowered and included a heterogeneous group of patients in terms of both disease severity and time of onset [12]. Therefore, it remains unclear if TPE offers a survival benefit in patients with septic shock [13].

Recently, our group has demonstrated in an uncontrolled study that TPE, applied as an adjunctive treatment in patients with early (< 24 h (hrs) since shock onset) and severe (norepinephrine (NE) dose > 0.4 μg/kg/min) septic shock, was associated with a rapid and significant reduction in catecholamine requirement [9]. Employing the same inclusion criteria of early and severe septic shock, we then performed a bi-center RCT comparing adjunctive TPE to standard of care (SOC). The primary endpoint, which showed a median reduction in NE requirement by almost 50% within 6 h, and key secondary endpoints of this trial have been reported earlier [14]. Since only limited findings were described in the format of a short letter, here we report the full set clinical and biochemical endpoints of this study. Additionally, we performed a multivariate mixed effects analysis of the primary endpoint NE reduction in order to identify patients that have benefited most from adjunctive TPE. This additional analysis might enable more precise designing of future large RCT investigating TPE in septic shock.

## Methods

### Study population

**Fig. 1:**
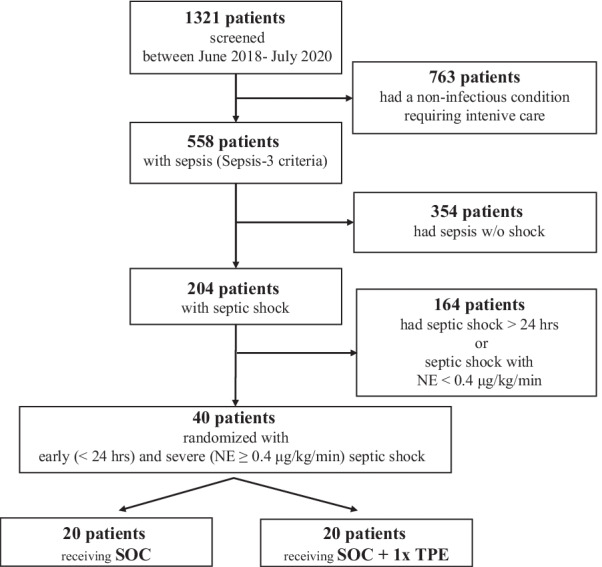
Flowchart of study participants. Shown are screening, enrollment and randomization of patients. Inclusion criteria were early (< 24 h) and severe (norepinephrine (NE) dose ≥ 0.4 μg/kg/min despite adequate fluid resuscitation) septic shock. The study compared standard of care (SOC) to SOC + a singular therapeutic plasma exchange (TPE), performed immediately following 1:1 envelope-based randomization

This was a prospective bi-center open-label randomized controlled trial at the Medical School Hannover and the University Hospital of Bonn, Germany. We screened *n* = 1321 patients admitted to the intensive care units (ICUs) of both hospitals from June 2018 to July 2020 for the presence of septic shock per SEPSIS-3 definition and the below explained in-/and exclusion criteria [1] (Fig. 1). All patients were treated according to the 2012 Surviving Sepsis Campaign (SSC) guidelines [15]. The ethical committee of Hannover Medical School (No. 2786-2015 and No. 8852_MPG_23b_2020) and University Hospital Bonn (No. 024/20) approved the protocol and written informed consent was obtained from participants or authorized representatives. The study was performed in accordance with the ethical standards laid down in the 1964 Declaration of Helsinki and its later amendments. The study was registered at clinicaltrials.gov (Identifier: NCT04231994).

### Inclusion and non-inclusion criteria

Patients were included based on: (1) septic shock with (2) onset of vasopressor use < 24 h prior to screening, and (3) profound systemic hypotension requiring norepinephrine (NE) doses of ≥ 0.4 µg/kg/min despite adequate intravenous fluid resuscitation (≥ 30 ml/kg bodyweight crystalloids). TPE had to be performed within 6 h after the randomization process. As exclusion criteria, we defined pregnancy or breast feeding, age < 18 years, end-stage chronic disease, and presence of a directive to withhold life-sustaining treatment.

### Therapeutic plasma exchange

Vascular access was established by central venous insertion of an 11-French two-lumen hemodialysis catheter. Based on previous experiences only a single TPE session was performed, since hemodynamic improvements were only achieved by the very first exchange [16]. TPE was performed against fresh frozen plasma (FFP), exchanging a fixed dose of 12 units of plasma (3262 ± 350 ml equal to 1 ± 0.3 times plasma volume) within 121 ± 37 min treatment time. Individual patient’s plasma volume was calculated in retrospect by a formula using patient weight and hematocrit [17]. In the majority (18/20) of patients a centrifugal TPE device (Spectra Optia Apheresis System) was used. Anticoagulation during TPE was achieved by regional citrate infusion. In patients with acute kidney injury (AKI), renal replacement therapy (RRT) was interrupted for the duration of TPE. Blood samples were drawn at randomization and 6 h following randomization. Patients were closely followed for the next 28 days, and survival was recorded. NE dose was titrated every 10–15 min to maintain a mean arterial pressure (MAP) above 65 mmHg.

### Endpoints

The primary endpoint was early hemodynamic improvement, indicated by absolute and relative NE reduction between randomization and 6 h following randomization.

Clinical secondary endpoints were the following: NE reduction between randomization and 24 h following randomization; reduction in the vasoactive-inotropic score (VIS) [18] between randomization and 6 h as well as after 24 h; Mean SOFA score over the first 9 days and 28-day mortality; Arterial lactate concentration, pO_2_/FiO_2_ ratio, total fluid balance, stroke volume variation (SVV), global end-diastolic volume index (GEDI), extravascular lung water index (ELWI), systemic vascular resistance index (SVRI), cardiac index (CI), all between randomization and 6 h thereafter; free days of vasopressors, mechanical ventilation, renal replacement therapy (RRT) and ICU within the first 28 days.

Biochemical secondary endpoints were absolute and relative change of procalcitonine (PCT), antithrombin-III (AT-III), protein C, a disintegrin and metalloproteinase with a thrombospondin type 1 motif, member 13 (ADAMTS13) activity, von Willebrand Factor Antigen (vWF:Ag), Angiopoietin-2 (Angpt-2) as well as soluble angiopoietin receptor (sTie-2), all between randomization and 6 h after randomization.

### Statistical analysis

Data were presented as median (25–75% IQR). Two-tailed p values of less than 0.05 were considered to indicate statistical significance. Comparisons of population characteristics between the TPE and the SOC group were performed using *t*-tests, Wilcoxon signed-rank tests and *χ*^2^ test, as appropriate. In order to compare the effect of TPE against SOC across the initial 24 h, paired *t*- and paired Wilcoxon signed-rank tests were employed after assessment for normality.

Survival data were analyzed by means of Cox proportional-hazards models as well as log-rank tests.

Modeling of the effect of TPE on repeated-measures of NE and lactate levels was approached by means of a linear mixed-effects model. NE (and lactate) measures were entered as outcome variable into the model, whereas TPE or SOC and time were entered as independent fixed effects including the interaction between both, finally per patient random intercepts were entered into the model. *P* values for individual fixed effects were obtained by Satterthwaite’s degrees of freedom method. In order to explore predictor variables for TPE effect, these were entered as additional fixed effects including a triple interaction term with TPE/ SOC and time, as well as all simple interaction terms between fixed effects. Model fit was assessed using a likelihood ratio test of the full model with the effects in question against a “null model”. Interaction terms were retained only if they were found to contribute to the model.

Statistical analysis was performed using GraphPad Prism 7 (La Jolla, CA), SPSS Statistics (IBM) and the R environment for statistical computing version 4.1.2 (R Foundation for Statistical Computing, Vienna, Austria).

## Results

### Cohort characterization

Based on the strict criteria we included 40 out of 1321 initially screened patients admitted to two tertiary care hospital ICUs (Fig. 1).

The demographic and clinical details are summarized in Table 1 demonstrating that both groups were comparable at randomization. Approximately, 80% of the patients were men with a median age around 55 years. The most common comorbidities were hypertension, obesity and diabetes. Pulmonary and abdominal infections were the most common cause of sepsis. In approximately 80% of patients, a causative pathogen, mostly gram + and gram- bacteria was identified and all patients were treated with a combination of broad-spectrum antibiotics. The median [IQR] SOFA score at inclusion was 16 [14–19] highlighting the degree of multi-organ failure in the overall cohort. The median NE dose was 0.6 μg/kg/min, significantly higher than required for study inclusion (≥ 0.4 μg/kg/min). Ninety-three percent of patients were mechanical ventilated due to respiratory failure and acute kidney injury (AKI) with need for renal replacement therapy (RRT) was present in 65% of the patients at inclusion. Despite continuous RRT and high dose vasopressor support, median lactate concentrations of 4 (2.6–6.1) mmol/l were detected. Markedly increased values for C-reactive protein (CRP), procalcitonine (PCT) and white blood cell count (WBC) were observed at randomization. In the TPE and the SOC group 17/20 and 20/20 patients received continuous corticosteroid medication within the first seven days since randomization (*p* = 0.722), respectively. At inclusion, 13/20 patients in the TPE group and 18/20 patients in the SOC group received continuous corticosteroids (*p* = 0.499). Corticosteroid preparation of choice was hydrocortisone given as a continuous intravenous drip at a dose of 200–240 mg/d (16/20 in the TPE and 17/20 in the SOC group) continued until shock resolution or death.

**Table 1:** Demographic and clinical parameters at study inclusion

Category	All *n* = 40	SOC *n* = 20	TPE *n* = 20	*p*
Age—years	56 (47–63)	57 (46–65)	55 (48–60)	0.663
Sex—no (%)				0.429
Male	32 (80)	17 (85)	15 (75)	
Female	8 (20)	3 (15)	5 (25)	
BMI—kg/m^2^	25.4 (22.6–32.3)	25.5 (24.1–35.4)	25.1 (20.2–31.1)	0.114
*Comorbidities—no (%)*				
Obesity	12 (30)	6 (30)	6 (30)	1
Hypertension	17 (42.5)	9 (45)	8 (40)	0.749
Diabetes	6 (15)	5 (25)	1 (5)	0.077
COPD	4 (10)	3 (15)	1 (5)	0.292
CHF	7 (17.5)	4 (20)	3 (15)	0.677
CAD	4 (10)	2 (10)	2 (10)	1
CKD	7 (17.5)	4 (20)	3 (15)	0.677
Immunosuppression	8 (20)	3 (15)	5 (25)	0.429
SOT or HSCT	5 (12.5)	3 (15)	2 (10)	0.633
*Sepsis onset—no (%)*				
Ambulatory	26 (65)	13 (65)	13 (65)	1
Hospital	14 (35)	7 (35)	7 (35)	1
*Side of infection—no (%)*				
Pulmo	25 (62.5)	12 (60)	13 (65)	0.744
Abdomen	12 (30)	6 (30)	6 (30)	1
Soft tissue	2 (5)	2 (10)	0 (0)	0.147
Endocarditis	1 (2.5)	0 (0)	1 (5)	0.311
*Identified pathogen—no (%)*				
Gram +	12 (30)	6 (30)	6 (30)	1
Gram-	12 (30)	5 (25)	7 (35)	0.49
Fungi	2 (5)	1 (5)	1 (5)	1
Viral	3 (7.5)	2 (10)	1 (5)	0.548
Mixed	2 (5)	2 (10)	0 (0)	0.147
Non-identified	9 (22.5)	4 (20)	5 (25)	0.705
SOFA score (points)	16.5 (14–19)	18 (14–20)	16 (13–18)	0.125
Norepinephrine dose (μg/kg/min)	0.591 (0.468–0.84)	0.582 (0.458–0.84)	0.598 (0.549–0.867)	0.724
VIS (points)	61 (48–85)	61 (46–85)	60 (55–87)	0.98
Mechanical ventilation—no (%)	37 (92.5)	18 (90)	19 (95)	0.548
Oxygenierungsindex (PaO_2_/FiO_2_)	145 (97–243)	156 (81–221)	132 (98–278)	0.624
*ECMO—no (%)*				
vv-ECMO	9 (22.5)	4 (20)	5 (25)	0.705
va-ECMO	2 (5)	2 (10)	0 (0)	0.147
Renal replacement therapy—no (%)	26 (65)	14 (70)	12 (60)	0.507
Lactate—mmol/l	4 (2.6–6.1)	4.4 (2.6–6.9)	4 (2.6–5.9)	0.513
*Organ failure—no (%)*				
Respiratory	39 (97.5)	19 (95)	20 (100)	0.311
Coagulation	19 (47.5)	10 (50)	9 (45)	0.752
Liver	16 (40)	11 (55)	5 (25)	0.053
Cardiovascular	40 (100)	20 (100)	20 (100)	1
Neurological	39 (97.5)	20 (100)	19 (95)	0.311
Renal	32 (80)	16 (80)	16 (80)	1
CRP (mg/l)	297 (168–350)	279 (115–410)	297 (213–350)	0.698
PCT (μg/l)	30 (7–82)	36 (13–101)	20 (6–59)	0.159
WBC (10^3^/μl)	17 (8–20)	12 (5–18)	18 (10–23)	0.244

Shown are both demographic and clinical characteristics at randomization for patients receiving standard of care treatment (SOC) as well as patients receiving additive therapeutic plasma exchange (TPE)

*BMI* body mass index, *CAD* coronary artery disease, *CHF* congestive heart failure, *CKD* chronic kidney disease, *COPD* chronic obstructive pulmonary disease, *CRP* C-reactive protein, *ECMO* extracorporeal membrane oxygenation (*vv* venovenous, *va* venoarterial), *HSCT* hematopoietic stem cell transplant, *NE* norepinephrine, *PCT* procalcitonine, *RRT* renal replacement therapy, *SOFA* Sequential Organ Failure Assessment, *SOT* solid organ transplant, *VIS* vasoactive-inotropic score, *WBC* white blood cell count

### Clinical endpoints

The primary endpoint has been presented in a short report recently [14]. In summary, the NE dose in the SOC group did not change within 6 h, but the NE dose decreased significantly in the TPE group by 48% (summarized in Table 2).

**Table 2:** Primary and secondary clinical outcomes

	Category	SOC					TPE					*p* between groups			
			0 h	6 h	24 h	*p*		0 h	6 h	24 h	*p*		0 h	6 h	24 h
Primary	NE dose (μg/kg/min)		0.582 (0.458–0.84)	0.482 (0.363–0.835)	0.362 (0.244–0.763)	0.052		0.598 (0.549–0.867)	0.335 (0.208–0.444)	0.183 (0.067–0.337)	< 0.0001	–	0.626	0.004	0.012
	Absolute ΔNE dose 0–6 h (μg/kg/min)	− 0.08 (− 0.242 to 0.06)	–	–	–	–	− 0.317 (− 0.554 to − 0.133)	–	–	–	–	0.003	–	–	–
	Relative ΔNE dose 0–6 h (%)	− 9.7 (− 31.7 to 13.8)	–	–	–	–	− 47.5 (− 71.6 to − 26.1)	–	–	–	–	0.001	–	–	–
Secondary	Absolute ΔNE dose 0–24 h (μg/kg/min)	− 0.121 (− 0.306 to 0.095)	–	–	–	–	− 0.463 (− 0.725 to − 0.314)	–	–	–	–	0.001	–	–	–
	Relative ΔNE dose 0–24 h (%)	− 24 (− 63.2 to 11)	–	–	–	–	− 71.5 (− 89 to − 58.4)	–	–	–	–	< 0.0001	–	–	–
	VIS Score (points)	–	61 (46–85)	62 (41–146)	37 (24–120)	0.227	–	60 (55–87)	31 (21–43)	23 (13–38)	< 0.0001	–	0.698	< 0.0001	0.028
	Absolute ΔVIS Score 0–6 h (points)	− 4 (− 24 to 37)	–	–	–	–	− 39 (− 58 to − 16)	–	–	–	–	0.0001	–	–	–
	Relative ΔVIS Score 0–6 h (%)	− 6 (− 36.4 to 39.5)	–	–	–	–	− 53.6 (− 73.4 to − 30.7)	–	–	–	–	< 0.0001	–	–	–
	Absolute ΔVIS Score 0–24 h (points)	− 14 (− 31 to 61)	–	–	–	–	− 42 (− 62 to − 29)	–	–	–	–	0.003	–	–	–
	Relative ΔVIS Score 0–24 h (%)	− 25.9 (− 63.2 to 62)	–	–	–	–	− 70.7 (− 78.7 to − 53.6)	–	–	–	–	0.002	–	–	–
	Mean SOFA Score d1-9 (points)	19 (15–24)	–	–	–	–	17 (12–21)	–	–	–	–	0.194	–	–	–
	28-day Mortality (%)	10/20 (50)	–	–	–	–	8/20 (40)	–	–	–	–	0.437	–	–	–
	Lactate (mmol/l)	–	4.4 (2.6–6.9)	4.3 (2.1–6.1)	3.1 (1.9–6.7)	0.628	–	4 (2.6–5.9)	4.1 (2.1–5.9)	1.7 (1.3–3.1)	0.014	–	0.644	0.664	0.055
	pO2/FiO2 (mmHg)	–	165 (74–227)	133 (100–205)	163 (91–216)	0.925	–	132 (98–278)	158 (125–241)	146 (119–259)	0.833	–	0.658	0.551	0.891
	Fluid balance (ml)	–	–	+ 1720 (867–2581)	+ 3675 (1129–6591)	0.025	–	–	+ 2048 (567–3054)	+ 3903 (294–5408)	0.008	–	–	0.682	0.452
	SVV (%)	–	15 (12–23)	16 (14–23)	–	0.48	–	17 (12–23)	10 (8–16)	–	0.135	–	0.908	0.069	–
	GEDI (ml/m2)	–	770 (650–955)	777 (710–1004)	–	0.501	–	718 (599–788)	712 (666–937)	–	0.186	–	0.127	0.509	–
	ELWI (ml/kg)	–	12 (7–19)	15 (8–21)	–	0.14	–	11 (9–19)	12 (9–16)	–	0.277	–	0.991	0.43	–
	SVRI (dyn*s*cm-5*m2)	–	1374 (895–1762)	1395 (952–2148)	–	0.122	–	1432 (1229–1691)	1171 (882–1374)	–	0.193	–	0.521	0.563	–
	CI (l/min/m2)	–	3.6 (2.2–4.6)	3.5 (2.3–4.7)	–	0.818	–	3.1 (2.8–4.1)	3.6 (3.2–4)	–	0.31	–	0.484	0.921	–
	Vasopressor free days (days)	11 ± 11	–	–	–	–	11 ± 10	–	–	–	–	0.976	–	–	–
	Ventilator free days (days)	10 ± 5	–	–	–	–	6 ± 8	–	–	–	–	0.209	–	–	–
	RRT free days (days)	7 ± 11	–	–	–	–	10 ± 12	–	–	–	–	0.491	–	–	–
	ICU free days (days)	4 ± 6	–	–	–	–	3 ± 5	–	–	–	–	0.512	–	–	–

Shown are primary and secondary clinical outcomes for patients receiving standard of care treatment (SOC) as well as patients receiving additive therapeutic plasma exchange (TPE). Endpoints are compared both longitudinally at 0, 6 and 24 h following randomization as well as between SOC and TPE groups

*CI* cardiac index, *ELWI* extravascular lung water index, *GEDI* global end-diastolic index, *ICU* intensive care unit, *MAP* mean arterial pressure, *NE* norepinephrine, *RRT* renal replacement therapy, *SOFA* Sequential Organ Failure Assessment, *SVV* stroke volume variation, *SVRI* systemic vascular resistance index, *VIS* vasoactive-inotropic score

Analyzing the long-term effects, we observed a preservation of this early effect even 24 h after randomization. While NE dose was 0.36 [0.24–0.76] µg/kg/min in the SOC group, it was 0.18 [0.07–0.34] µg/kg/min in the TPE group (*p* = 0.01, Table 2). This corresponded to an absolute NE dose reduction of − 0.12 µg/kg/min in the SOC group compared to − 0.46 µg/kg/min in the TPE group (*p* = 0.001, Table 2); the relative median NE dose reduction at 24 h compared to baseline was − 24 [− 63 to + 11) % for control patients compared to − 72 [− 89 to − 58] % for TPE treated patients (*p* < 0.0001, Table 2). Absolute NE dose of survivors was not different at 48 (*p* = 0.495) and 72 h (*p* = 0.281) following randomization (data not shown).

To additionally investigate the effect of TPE on hemodynamics if further vasopressors (e.g., argipressin) as well as additional inotropes were required, we compared the VIS, which quantitatively summarizes cumulative doses of vasopressors and inotropes applied [18], between groups. The VIS was unchanged in the SOC group at 6 h following randomization (VIS: 61 [46–85] vs 62 [41–146] points, *p* = 0.984, Table 2). In contrast, in the TPE group the VIS was reduced by half (60 [55–87] vs 31 [20–43] points, *p* < 0.0001, between-group difference at 6 h: *p* < 0.0001, Table 2). At 24 h following randomization, a significant difference between groups remained (*p* = 0.028, Table 2).

Consistent with reduction in NE, lactate concentration showed a significant decline in the TPE group within 24 h after randomization (*p* = 0.014), which was not found in the SOC group (*p* = 0.628, Table 2). Total fluid balances increased in both groups during the first 24 h and were not different between groups. However, stroke volume variation (SVV), a dynamic measure of preload, remained unchanged in the SOC group while it numerically decreased in the TPE group (*p* = 0.069 between-group difference at 6 h, Table 2). All other parameters measured by PiCCO (measured in a subgroup of 13 patients in the SOC and 11 in the TPE group) monitoring showed no differences between groups (Table 2).

Although numerically lower in the TPE group, neither the mean SOFA score over the first 9 days, nor the 28-day mortality was significantly different between groups [14]. Early mortality after 48 h following randomization was 30% in the SOC and 10% in the TPE group (*p* = 0.095). Patients with pulmonary focus of infection had a 28-day mortality of only 15% in the TPE group while it was 42% in the SOC group (HR 0.297 [0.057–1.538], Cox regression *p* = 0.148, log-rank test *p* = 0.095). In contrast, mortality of patients with an abdominal focus of infection was high in both groups (67 vs 83%, HR 1.575 [0.411–6.034], Cox regression *p* = 0.507). Patients in both groups had a comparable extent of total free days of ventilator, vasopressors, RRT and ICU during a 28-day period since randomization (Table 2).

### Biochemical endpoints

**Fig. 2:**
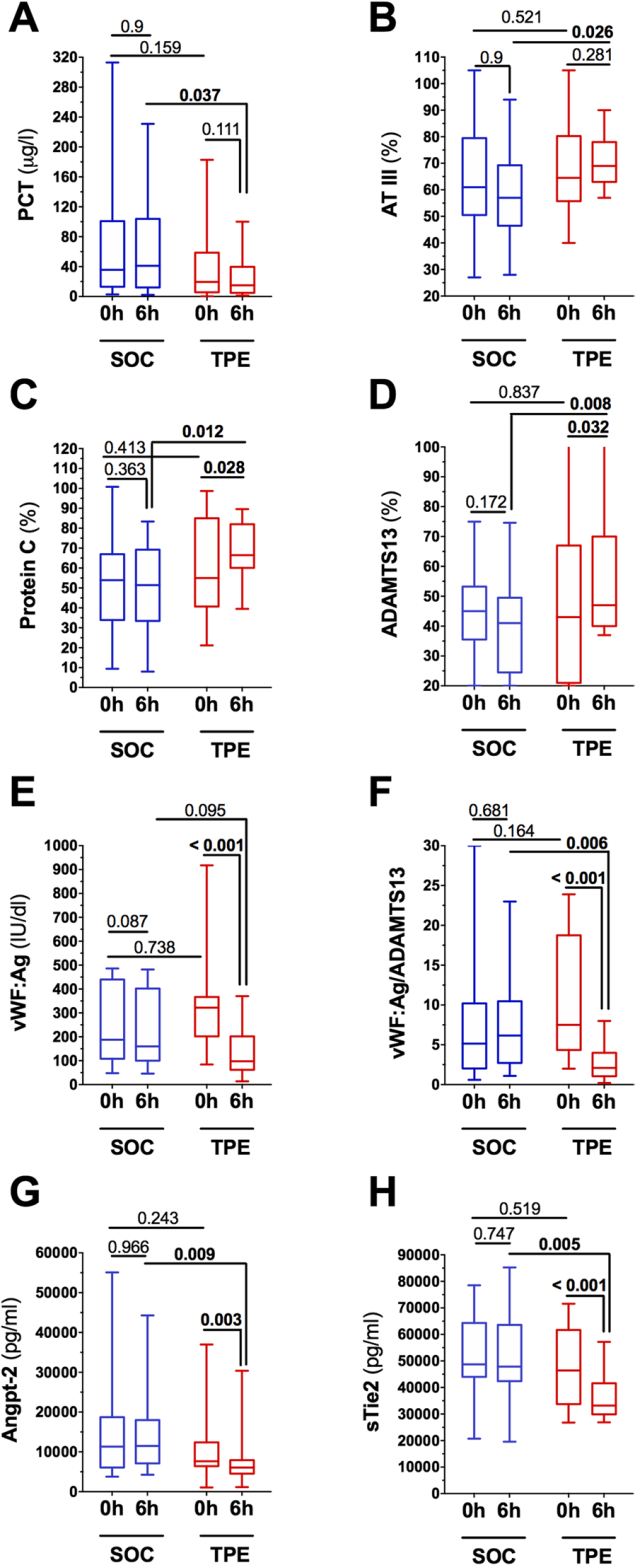
Secondary biochemical endpoints. Box and whisker blots showing **A** Procalcitonin (PCT), **B** Antithrombin-III (AT-III), **C** Protein C, **D** A disintegrin and metalloprotease with thrombospondin-1-like domains 13 (ADAMTS13), **E** von Willebrand factor antigen (vWF:Ag), **F** vWF:Ag/ADAMTS13 ratio, **G** Angiopoietin-2 (Angpt-2), **H** soluble receptor of tyrosine kinase with immunoglobulin-like and EGF-like domains (sTie-2) for patients receiving standard of care (SOC) treatment as well as patients receiving additive therapeutic plasma exchange (TPE). Compared are results both at randomization and 6 h after randomization and between-group differences

PCT further increased in the SOC group, while it was reduced in the TPE group (PCT at 6 h after randomization: 41.1 [12.2–103.9] vs 15 [4.9–39.7] μg/l, *p* = 0.037, Fig. 2A).

Antithrombin (AT)-III increased in the TPE but not in the SOC group (AT-III at 6 h after randomization: 57 [47–69] vs 69 [63–78] %, *p* = 0.026, Fig. 2B). The same observation was made for protein C (protein C at 6 h after randomization: 51 [27–67] vs 67 [60–82] %, *p* = 0.012, Fig. 2C). ADAMTS-13 activity was unchanged in the SOC group, while it increased significantly in the TPE group C (ADAMTS-13 at 6 h after randomization: 41 [25–50] vs 47 [40–70] %, *p* = 0.008, Fig. 2D). In contrast, vWF:Ag was profoundly reduced in the TPE group (vWF:Ag for TPE group at baseline vs 6 h after randomization: 322 [202–367] vs 98 [62–202] %, *p* < 0.0001, Fig. 2E), an effect not seen in the SOC group. Consequentially, the ratio of vWF:Ag to ADAMTS-13 activity was unchanged in the SOC group but significantly decreased in the TPE group (vWF:Ag/ADAMTS-13 at 6 h after randomization: 6.2 [2.7–10.5] vs 2.1 [1.1–4], *p* = 0.006, Fig. 2F).

Angpt-2 concentration remained stable elevated in the SOC group but could be reduced in the TPE group (Angpt-2 at 6 h after randomization: 11.49 [7.1–18.0] vs 6.1 [4.5–7.9] ng/ml, *p* = 0.009, Fig. 2G). The same effect of TPE was observed for sTie-2 (sTie-2 at 6 h after randomization: 47.8 [42.4–63.6] vs 33.2 [29.9–41.6] ng/ml, *p* = 0.005, Fig. 2H).

### Prediction of NE dose response and lactate levels over 24 h in a linear model

**Fig. 3:**
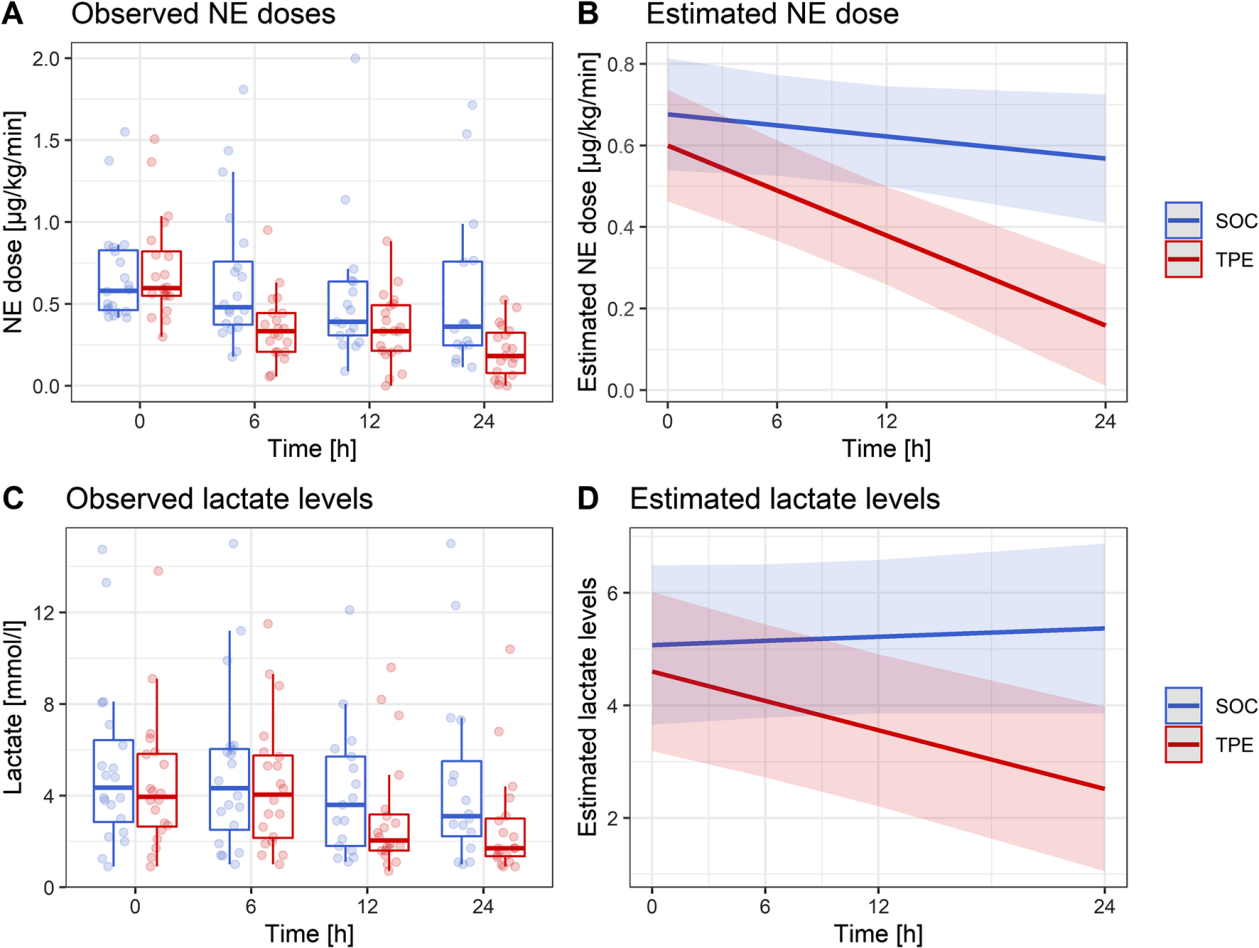
Modulation of TPE effect on norepinephrine dose and lactate concentrations. Shown are both observed **A, C** and estimated **B**, **D** norepinephrine doses (NE) as well as lactate concentrations for the standard of care (SOC) and therapeutic plasma exchange (TPE) group during the first 24 h since randomization. Estimated values were calculated using a linear mixed-effects model. The models indicated a continuous effect of TPE on the reduction in NE doses (*p* = 0.004) and lactate concentrations (*p* = 0.001) over the initial 24 h

A mixed-effect model (Additional file 1: Table S1) indicated a continuous effect of TPE on the reduction in NE doses over the initial 24 h. As opposed to the SOC group, which presented an estimated NE dose reduction of 0.005 µg/kg/min per hour, patients in the TPE group experienced an estimated NE reduction of 0.018 µg/kg/min per hour (*p* = 0.004) (Fig. 3A, B).

Similarly, under TPE serum lactate levels continuously decreased over the initial 24 h whereas they increased under SOC (*p* = 0.001) (Fig. 3 C, D & Additional file 1: Table S2).

**Fig. 4:**
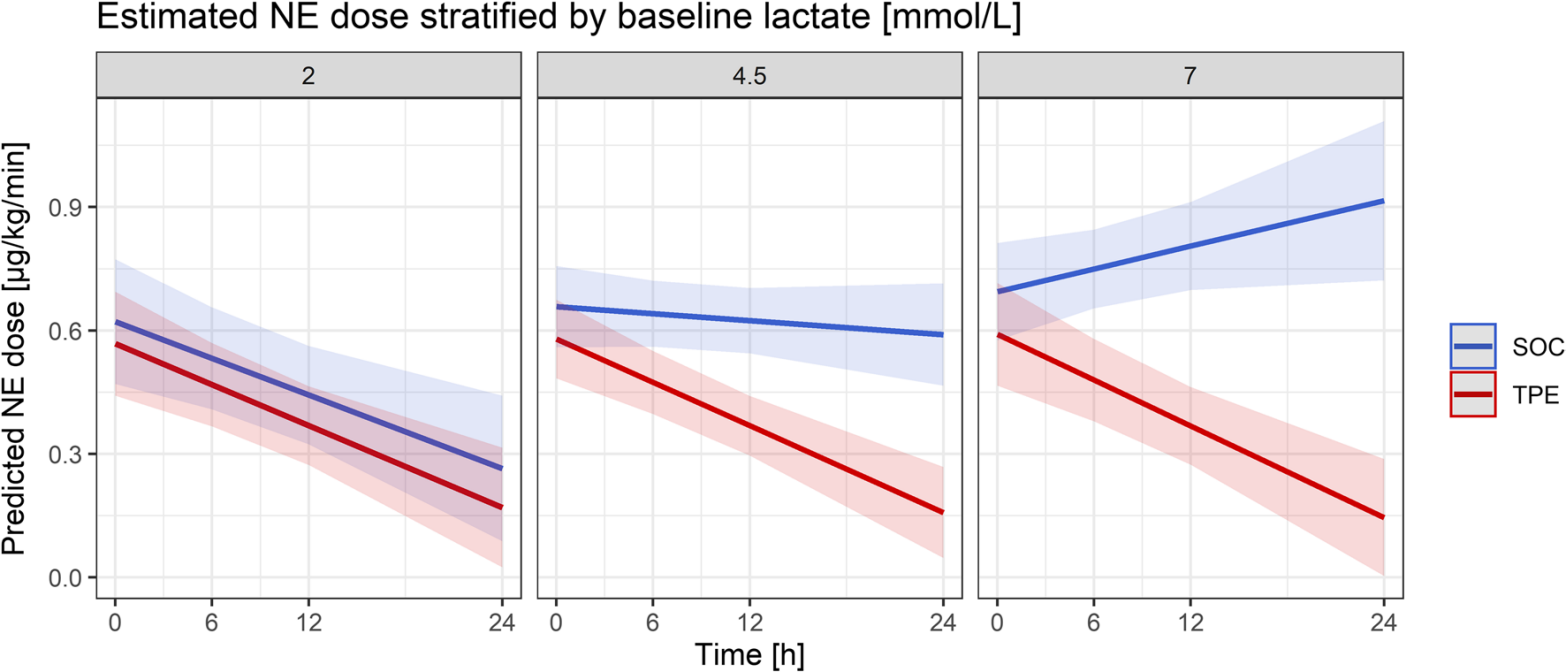
Prediction of effect of TPE on norepinephrine dose by baseline lactate concentration. Shown are estimated norepinephrine (NE) doses for both the standard of care (SOC) and therapeutic plasma exchange (TPE) group stratified by different lactate concentrations at randomization. Estimated values were calculated using a triple interaction model with TPE/ SOC and time, as well as all simple interaction terms between fixed effects. The model indicated that patients with increasing baseline lactate levels experienced diminishing NE dose reductions over 24 h when under SOC, in contrast to patients under TPE which experienced sustained NE reductions across all levels of lactate (*p* = 0.004). At baseline lactate concentrations of 2 mmol/l, both groups showed a reduction in NE (left panel). Above 4.5 mmol/l, patients under SOC experienced no NE dose reduction, whereas NE reduction in the TPE group remained conserved (middle panel). Above 7 mmol/l, patients in the SOC group showed increasing NE doses over time, while NE reduction was conserved in the TPE group (right panel). The thresholds for baseline lactate concentration employed were chosen post hoc in order to best illustrate the continuous effect of lactate within the model

Solely baseline lactate levels were found to be predictive for the effect of TPE on NE reduction over the initial 24 h (Additional file 1: Table S3). Patients with increasing baseline lactate levels experienced diminishing NE dose reductions over 24 h when under SOC, in contrast to patients under TPE which experienced sustained NE reductions across all levels of lactate (*p* = 0.004). Thus, above approximately 3 mmol/l of lactate the slopes of NE dose reduction between TPE and SOC became disjoint, and above 4.5 mmol/l patients under SOC experienced no NE dose reduction, whereas NE reduction in the TPE group remained conserved (Fig. 4).

## Discussion

This prospective randomized bicentric trial shows that early TPE in patients with septic shock leads to hemodynamic stabilization. The primary endpoint showed a reduction in NE after 6 h following randomization of about 50% found in the TPE group. This early hemodynamic stabilization was also applicable for additional vasoactive and inotropic agents used (indicated by the VIS), was preserved 24 h after randomization and was accompanied by a reduction in blood lactate indicating shock reversal in the TPE group. Although NE dose was not different between groups beyond 24 h, this effect could be confounded by a higher mortality in the SOC group within the first 48 h after randomization. These results confirm earlier findings from non-randomized trials [9, 19]. Although total fluid balance was not different between both groups, SVV was improved in the TPE cohort, which might indicate additional greater intravascular filling following TPE treatment. In accordance with this hypothesis, improved fluid balances have been observed repeatedly with additive TPE treatment [9, 19].

More recently, distinct biological and clinical sepsis phenotypes have been identified that might respond differently to specific therapeutic measures [20]. As such, one of the major challenges in future precision medicine orientated sepsis therapy will lie in the correct identification of subgroups that might benefit most from a certain additive therapy modality. Two robust mixed-effect models indicated continuous effects of TPE on the reduction in both NE doses and lactate concentration over the initial 24 h following randomization. Overall, this might indicate improvements in both macro- and microcirculatory dysfunction associated with TPE treatment. Furthermore, when several baseline variables were explored as potential predictor variables for TPE effect on NE dose utilizing an additional multivariate mixed-effects model including a triple interaction term with TPE/SOC and time, only baseline lactate concentration turned out to be a significant predictor of later hemodynamic response to TPE. Patients with increasing baseline lactate levels experienced diminishing NE dose reductions over 24 h when under SOC, in contrast to patients under TPE, which experienced sustained NE reductions across all levels of lactate. Already above a baseline lactate above 3 mmol/l, the slopes of NE dose reduction between TPE and SOC became disjoint, and above 4.5 mmol/l patients under SOC experienced no NE dose reduction at all, whereas this remained conserved in the TPE group. This observation is of relevance as both absolute lactate and lactate clearance has been closely associated with survival in patients with septic shock [21]. That TPE appears to be effective in initiating both hemodynamic stabilization and lactate clearance and is especially beneficial in terms of hemodynamic improvement in patients with initial high lactate concentrations is a promising finding. Baseline lactate concentration might also facilitate further stratification in future larger prospective studies investigating TPE in septic shock patients.

In contrast to other modalities of adjunctive extracorporeal sepsis treatment [7], TPE has the potential to not only remove excessive potentially injurious mediators (e.g., cytokines) but also to replace depleted protective factors that can be found in physiological concentrations within the healthy donor plasma used for the exchange [6]. Consistently, we observed with TPE a reduction in injurious mediators such as PCT, vWF:Ag, Angpt-2, sTie-2 and a repletion of decreased protective factors such as AT-III, Protein C, ADAMTS-13, which were not seen in the control group. These results confirm previously made observations from non-randomized investigations [8, 9].

Increased PCT concentrations have been closely associated with reduced survival in both preclinical sepsis models [22, 23] and clinical observations [24]. Some data indicate that PCT is not only a biomarker but might also be a disease mediator making it promising as a potential therapeutic target [22]. Therefore, the observation that a circulating marker/meditator like PCT could be lowered after TPE to half of the control group level is of potential importance.

Supplementation of septic patients with AT-III has been investigated for a long time and did not show a mortality benefit in early trials involving heterogeneous patient populations [25]. It might, however, reduce mortality in selected highly unstable septic patients, including those with disseminated intravascular coagulation (DIC) [26–28]. Median ISTH-DIC score in our study was 4 (3–5) with elevated D-Dimers of 7.9 (3–18.2) mg/l, indicating non-overt DIC in the overall cohort with overt DIC in some patients. Depletion of the endogenous anticoagulation factor “protein C” by increased consumption, degradation, and/or decreased synthesis, is a well-described characteristic of sepsis and has been shown to predict mortality in sepsis [29]. Interestingly, substitution of its activated form termed Drotrecogin alfa improved 28-day survival in the initial randomized controlled trial [30], but failed to reproduce these findings in a later confirmatory study of patients with septic shock [31]. Heterogeneity of distinct biological and clinical sepsis phenotypes that might respond different to specific therapeutic measures such as protein C supplementation might in part explain these conflicting results [20]. Particularly, severe deficiency of protein C (e.g., in purpura fulminans) is associated with inferior outcomes in sepsis [29] and treatment options, including protein C supplementation, are continued to be evaluated in these selected patients [32, 33]. Median protein C activity of our cohort at randomization was 54.5 (39.8–70) %, indicating protein C deficiency to a certain extend in all patients. Of note, TPE did increase AT-III activity.

Severe ADAMTS13 deficiency (the VWF cleaving protease) causes accumulation of ultra-large vWF multimers (ULVWF) that can lead to the clinical picture of thrombotic microangiopathy as seen in its most severe form in thrombotic thrombocytopenic purpura (TTP) [34]. Interestingly, a deficiency of ADAMTS13 is also detectable in sepsis [35–37]. At the same time, large amounts of vWF:Ag are secreted by the activated septic endothelium leading to both increased platelet aggregation and formation of highly pro-thrombotic ULVWF multimers [38]. Consequentially, an increased vWF:Ag/ADAMTS13 ratio has been repeatedly associated with severity of shock and organ failure as well as increased mortality in sepsis [35, 39–42]. Median ADAMTS13 activity was lowered to 43% with a relatively wide IQR of 25 to 55%. ADAMTS13 activity below 45% has already been associated with increased mortality in sepsis [41], activity below 30% with significantly higher systemic inflammation (i.e., IL-6 concentrations) [40] and greater incidence of overt DIC [36]. While we did not measure ULVWF multimers, vWF:Ag concentration was more than three times as high as normal at baseline but normalized in patients following a singular TPE treatment.

Angpt-2, a pre-stored protein secreted by stimulated endothelium and an antagonist of the vascular barrier protective receptor Tie2, contributes to the pathophysiology of septic multiple organ dysfunction [43–45]. Increased circulating Angpt-2 concentrations are associated with both organ failure and mortality in septic patients [46] and initial Angpt-2 concentrations below 9.2 ng/ml have been associated with favorable survival. Here, we show a significant reduction in circulating Angpt-2 following TPE to around 6 ng/ml, while it remains elevated in the control group. Finally, cleaved circulating receptor binding domain sTie2 has been demonstrated to locally inhibit endothelium protective Angpt-1 signaling by trapping protective Angpt-1 [47]. Of note, sTie2 circulating concentration was reduced by 30% following a singular TPE session.

This exploratory study was not powered to demonstrate a difference in organ dysfunction or mortality. Thus, although numerical trends were observed for the TPE group toward lower median SOFA Scores and 28-day mortalities, no statistically significant differences could be observed. Recent retrospective analyses have suggested lower degrees of organ dysfunction as well as mortality in septic shock patients following treatment with adjunctive TPE [19, 48]. In a recent propensity-score-matched retrospective analysis, patients with pneumonia as the primary site of infection demonstrated the greatest improvement in 28-day mortality by additive TPE [19]. Consistent with this observation we found that patients with a lung focus of infection had a better response to TPE with 28-day mortality of 15% compared to 42% in the control group. The observation that pulmonary sepsis foci had numerically better survival upon TPE is encouraging and deserves further analysis. Despite the recent acknowledgment of source-specific host responses in sepsis [49] where abdominal foci demonstrated stronger inflammatory patterns along with vascular permeability and coagulation compared to a pulmonary focus, observed beneficial TPE response in pneumonia might be influenced by relevant confounders such as source control, bacterial resistance, and prevalent comorbidities.

TPE has been investigated as an adjunctive treatment modality for sepsis earlier [9, 12, 19, 48, 50–52] with overall inconclusive results [11] preventing advice toward a routine use [53]. The informative value of these previous studies was limited by both a heterogeneity of the incorporated inclusion criteria (e.g., adult and pediatric patients, patients with and without shock, different durations of sepsis shock onset before inclusion) as well as the non-randomized nature of most studies. A major strength of this current trial therefore is a homogenization of the patient cohort investigated by only including patients with severe (NE dose > 0.4 μg/kg/min) and early (< 24 h since onset) septic shock. By assessing, in addition to the clinical data presented, a collection of (non-routinely measured) biochemical parameters, we suggest a possible pathophysiological explanation for the observed improved hemodynamic stabilization found following TPE.

TPE using FFP as replacement fluid has several potential adverse events, including infectious and non-infectious (allergic reaction, transfusion associated lung injury (TRALI), citrate toxicity, hypotension) [54] with pruritus and urticaria most commonly observed [55]. However, severe adverse events are rare [56] and incidence of adverse events requiring discontinuation of treatment lies at around 0.2% [55]. Of note, no adverse events were observed in this present patient cohort.

This study has important limitations, mainly its small sample size, preventing to draw conclusions about hard endpoints such as organ-dysfunction or mortality. In addition, the intervention was administered as a singular regimen and at a fixed dose, which precludes us from providing data on effects at different dosages or time frames. A fixed dose of exchanged plasma volume was preferred over a more conventional weight and hematocrit-based dose for reasons of simplicity of the protocol. A minority of patients therefore has been treated with plasma volumes lower than the general recommendation made by the American Society of Apheresis (AFSA) (1–1.5 times plasma volume) [13]. The absence of a third therapeutic arm testing plasma exchange with albumin as replacement fluid, prevents to draw conclusions concerning the underlying reason for the beneficial effects seen in terms of hemodynamic stabilization, e.g., due to removal of injurious mediators or replacement with protective factors. However, it might be possible that exactly the combination of both principles might be important for restoration of hemostasis in septic shock [57]. The use of lactate as a parameter to predict response to treatment was not proposed a-priori. Therefore, these results are hypothesis generating and meant to inform a larger follow-up study, which will be suitable to confirm (or falsify) these observations.

## Conclusions

Our explorative randomized study demonstrated improved hemodynamic stabilization and lactate clearance following adjunctive TPE in a subgroup of early septic shock patients. Higher baseline lactate concentrations predicted response to TPE and may guide future designs of a randomized, controlled multicenter study to further investigate this treatment modality.

## Supplementary Information

**Additional file1: Table S1**. Linear mixed effect model for the prediction of norepinephrine doses. **Table S2**. Linear mixed effect model for the prediction of serum lactate levels. **Table S3**. Linear mixed effect model for the prediction of the norepinephrine dose.

## Data Availability

The datasets used and analyzed are during the current study are available from the corresponding author on reasonable request.

## Abbreviations

ADAMTS13: A disintegrin and metalloprotease with thrombospondin-1-like domains 13
AFSA: American Society of Apheresis
AT-III: Antithrombin-III
BMI: Body mass index
CAD: Coronary artery disease
CKD: Chronic kidney disease
CHF: Congestive heart failure
CI: Cardiac index
COPD: Chronic obstructive pulmonary disease
CRP: C-reactive protein
ECMO: Extracorporeal membrane oxygenation (vv = venovenous, va = venoarterial)
ELWI: Extravascular lung water index
FFP: Fresh frozen plasma
GEDI: Global end-diastolic index
HSCT: Hematopoietic stem cell transplant
HR: Hazard ratio
ICU: Intensive care unit
IL: Interleukin
MAP: Mean arterial pressure
NE: Norepinephrine
OR: Odds ratio
PCT: Procalcitonine
RCT: Randomized controlled trial
RRT: Renal replacement therapy
SOC: Standard of care
SOFA: Sequential Organ Failure Assessment
SOT: Solid organ transplant
sTie2: A soluble receptor of tyrosine kinase with immunoglobulin-like and EGF-like domains 2
SVV: Stroke volume variation
SVRI: Systemic vascular resistance index
TPE: Therapeutic plasma exchange
ULVWF: Ultra-large vWF multimers
VIS: Vasoactive-inotropic score
vWF:Ag: Von Willebrand factor antigen
WBC: White blood cell count

## References

[1] Singer M, Deutschman CS, Seymour CW, Shankar-Hari M, Annane D, Bauer M (2016). The third international consensus definitions for sepsis and septic shock (sepsis-3). JAMA.

[2] Fleischmann C, Thomas-Rueddel DO, Hartmann M, Hartog CS, Welte T, Heublein S (2016). Hospital incidence and mortality rates of sepsis. Deutsches Arzteblatt Int.

[3] Angus DC, van der Poll T (2013). Severe sepsis and septic shock. N Engl J Med.

[4] Rhodes A, Evans LE, Alhazzani W, Levy MM, Antonelli M, Ferrer R (2017). Surviving sepsis campaign: international guidelines for management of sepsis and septic shock: 2016. Intensive Care Med.

[5] Steinhagen F, Schmidt SV, Schewe JC, Peukert K, Klinman DM, Bode C (2020). Immunotherapy in sepsis - brake or accelerate?. Pharmacol Ther.

[6] David S, Stahl K (2019). To remove and replace-a role for plasma exchange in counterbalancing the host response in sepsis. Crit Care (London, England).

[7] Stahl K, Bode C, David S (2021). Extracorporeal strategies in sepsis treatment: role of therapeutic plasma exchange. Anasthesiol Intensivmed Notfallmed Schmerzther AINS.

[8] Stahl K, Schmidt JJ, Seeliger B, Schmidt BMW, Welte T, Haller H (2020). Effect of therapeutic plasma exchange on endothelial activation and coagulation-related parameters in septic shock. Crit Care (London, England).

[9] Knaup H, Stahl K, Schmidt BMW, Idowu TO, Busch M, Wiesner O (2018). Early therapeutic plasma exchange in septic shock: a prospective open-label nonrandomized pilot study focusing on safety, hemodynamics, vascular barrier function, and biologic markers. Crit Care (London, England).

[10] Stahl K, Bikker R, Seeliger B, Schmidt JJ, Schenk H, Schmidt BMW (2020). Effect of therapeutic plasma exchange on immunoglobulin deficiency in early and severe septic shock. J Intensive Care Med.

[11] Rimmer E, Houston BL, Kumar A, Abou-Setta AM, Friesen C, Marshall JC (2014). The efficacy and safety of plasma exchange in patients with sepsis and septic shock: a systematic review and meta-analysis. Crit care (London, England).

[12] Busund R, Koukline V, Utrobin U, Nedashkovsky E (2002). Plasmapheresis in severe sepsis and septic shock: a prospective, randomised, controlled trial. Intensive Care Med.

[13] Schwartz J, Padmanabhan A, Aqui N, Balogun RA, Connelly-Smith L, Delaney M (2016). Guidelines on the use of therapeutic apheresis in clinical practice-evidence-based approach from the writing committee of the american society for apheresis: the seventh special issue. J Clin Apher.

[14] David S, Bode C, Putensen C, Welte T, Stahl K (2021). Adjuvant therapeutic plasma exchange in septic shock. Intensive Care Med.

[15] Dellinger RP, Levy MM, Rhodes A, Annane D, Gerlach H, Opal SM (2013). Surviving sepsis campaign: international guidelines for management of severe sepsis and septic shock, 2012. Intensive Care Med.

[16] David S, Hoeper MM, Kielstein JT (2017). Plasma exchange in treatment refractory septic shock : presentation of a therapeutic add-on strategy. Med Klinik Intensivmed Notfallmed.

[17] Kaplan AA (1990). A simple and accurate method for prescribing plasma exchange. ASAIO Trans.

[18] McIntosh AM, Tong S, Deakyne SJ, Davidson JA, Scott HF (2017). Validation of the vasoactive-inotropic score in pediatric sepsis. Pediatr Crit Care Med J Soc Crit Care Med World Fed Pediatr Intensive Crit Care Soc.

[19] Keith PD, Wells AH, Hodges J, Fast SH, Adams A, Scott LK (2020). The therapeutic efficacy of adjunct therapeutic plasma exchange for septic shock with multiple organ failure: a single-center experience. Crit Care (London, England).

[20] Seymour CW, Kennedy JN, Wang S, Chang CH, Elliott CF, Xu Z (2019). Derivation, validation, and potential treatment implications of novel clinical phenotypes for sepsis. JAMA.

[21] Haas SA, Lange T, Saugel B, Petzoldt M, Fuhrmann V, Metschke M (2016). Severe hyperlactatemia, lactate clearance and mortality in unselected critically ill patients. Intensive Care Med.

[22] Nylen ES, Whang KT, Snider RH, Steinwald PM, White JC, Becker KL (1998). Mortality is increased by procalcitonin and decreased by an antiserum reactive to procalcitonin in experimental sepsis. Crit Care Med.

[23] Becker KL, Snider R, Nylen ES (2010). Procalcitonin in sepsis and systemic inflammation: a harmful biomarker and a therapeutic target. Br J Pharmacol.

[24] Adamik B, Smiechowicz J, Jakubczyk D, Kübler A (2015). Elevated serum PCT in septic shock with endotoxemia is associated with a higher mortality rate. Medicine.

[25] Warren BL, Eid A, Singer P, Pillay SS, Carl P, Novak I (2001). Caring for the critically ill patient. high-dose antithrombin III in severe sepsis: a randomized controlled trial. JAMA.

[26] Hayakawa M, Kudo D, Saito S, Uchino S, Yamakawa K, Iizuka Y (2016). Antithrombin supplementation and mortality in sepsis-induced disseminated intravascular coagulation: a multicenter retrospective observational study. Shock (Augusta, Ga).

[27] Tagami T, Matsui H, Horiguchi H, Fushimi K, Yasunaga H (2014). Antithrombin and mortality in severe pneumonia patients with sepsis-associated disseminated intravascular coagulation: an observational nationwide study. J Thromb Haemost JTH.

[28] Wiedermann CJ, Hoffmann JN, Juers M, Ostermann H, Kienast J, Briegel J (2006). High-dose antithrombin III in the treatment of severe sepsis in patients with a high risk of death: efficacy and safety. Crit Care Med.

[29] Shorr AF, Bernard GR, Dhainaut JF, Russell JR, Macias WL, Nelson DR (2006). Protein C concentrations in severe sepsis: an early directional change in plasma levels predicts outcome. Crit care (London, England).

[30] Bernard GR, Vincent JL, Laterre PF, LaRosa SP, Dhainaut JF, Lopez-Rodriguez A (2001). Efficacy and safety of recombinant human activated protein C for severe sepsis. N Engl J Med.

[31] Ranieri VM, Thompson BT, Barie PS, Dhainaut JF, Douglas IS, Finfer S (2012). Drotrecogin alfa (activated) in adults with septic shock. N Engl J Med.

[32] Brunkhorst FM, Patchev V (2014). Sepsis-associated Purpura Fulminans International Registry-Europe (SAPFIRE). Medizinische Klinik, Intensivmedizin und Notfallmedizin.

[33] Knoebl P, Schellongowski P, Staudinger T, Sperr WR, Scheibenpflug C (2013). Treatment of infection-associated purpura fulminans with protein C zymogen is associated with a high survival rate. Blood.

[34] Levy GG, Nichols WC, Lian EC, Foroud T, McClintick JN, McGee BM (2001). Mutations in a member of the ADAMTS gene family cause thrombotic thrombocytopenic purpura. Nature.

[35] Aibar J, Castro P, Espinosa G, Fernández S, Hernández C, Rinaudo M (2015). ADAMTS-13 in critically Ill patients with septic syndromes and noninfectious systemic inflammatory response syndrome. Shock (Augusta, Ga).

[36] Bockmeyer CL, Claus RA, Budde U, Kentouche K, Schneppenheim R, Lösche W (2008). Inflammation-associated ADAMTS13 deficiency promotes formation of ultra-large von willebrand factor. Haematologica.

[37] Kremer Hovinga JA, Zeerleder S, Kessler P, Romani de Wit T, van Mourik JA, Hack CE (2007). ADAMTS-13, von Willebrand factor and related parameters in severe sepsis and septic shock. J Thromb Haemost JTH.

[38] Peetermans M, Meyers S, Liesenborghs L, Vanhoorelbeke K, De Meyer SF, Vandenbriele C (2020). Von Willebrand factor and ADAMTS13 impact on the outcome of staphylococcus aureus sepsis. J Thromb Haemost JTH.

[39] Ono T, Mimuro J, Madoiwa S, Soejima K, Kashiwakura Y, Ishiwata A (2006). Severe secondary deficiency of von Willebrand factor-cleaving protease (ADAMTS13) in patients with sepsis-induced disseminated intravascular coagulation: its correlation with development of renal failure. Blood.

[40] Peigne V, Azoulay E, Coquet I, Mariotte E, Darmon M, Legendre P (2013). The prognostic value of ADAMTS13 (a disintegrin and metalloprotease with thrombospondin type 1 repeats, member 13) deficiency in septic shock patients involves interleukin-6 and is not dependent on disseminated intravascular coagulation. Crit care (London, England).

[41] Lin JJ, Chan OW, Hsiao HJ, Wang Y, Hsia SH, Chiu CH (2016). Decreased ADAMTS 13 activity is associated with disease severity and outcome in pediatric severe sepsis. Medicine.

[42] Claus RA, Bockmeyer CL, Budde U, Kentouche K, Sossdorf M, Hilberg T (2009). Variations in the ratio between von Willebrand factor and its cleaving protease during systemic inflammation and association with severity and prognosis of organ failure. Thromb Haemost.

[43] Stiehl T, Thamm K, Kaufmann J, Schaeper U, Kirsch T, Haller H (2014). Lung-targeted RNA interference against angiopoietin-2 ameliorates multiple organ dysfunction and death in sepsis. Crit Care Med.

[44] David S, Mukherjee A, Ghosh CC, Yano M, Khankin EV, Wenger JB (2012). Angiopoietin-2 may contribute to multiple organ dysfunction and death in sepsis*. Crit Care Med.

[45] Kumpers P, Gueler F, David S, Slyke PV, Dumont DJ, Park JK (2011). The synthetic tie2 agonist peptide vasculotide protects against vascular leakage and reduces mortality in murine abdominal sepsis. Crit care (London, England).

[46] Kümpers P, Hafer C, David S, Hecker H, Lukasz A, Fliser D (2010). Angiopoietin-2 in patients requiring renal replacement therapy in the ICU: relation to acute kidney injury, multiple organ dysfunction syndrome and outcome. Intensive Care Med.

[47] Alawo DOA, Tahir TA, Fischer M, Bates DG, Amirova SR, Brindle NPJ (2017). Regulation of angiopoietin signalling by soluble Tie_2_ ectodomain and engineered ligand trap. Sci Rep.

[48] Aydin K, Korkmaz S, Erkurt MA, Sarici A, Ekinci O, Baysal NA (2021). Apheresis in patients with sepsis: a multicenter retrospective study transfusion and apheresis science: official journal of the world apheresis association: official. J Eur Soc Haemapheresis..

[49] Peters-Sengers H, Butler JM, Uhel F, Schultz MJ, Bonten MJ, Cremer OL (2022). Source-specific host response and outcomes in critically ill patients with sepsis: a prospective cohort study. Intensive Care Med.

[50] Reeves JH, Butt WW, Shann F, Layton JE, Stewart A, Waring PM (1999). Continuous plasmafiltration in sepsis syndrome. plasmafiltration in sepsis study group. Crit Care Med.

[51] Nguyen TC, Han YY, Kiss JE, Hall MW, Hassett AC, Jaffe R (2008). Intensive plasma exchange increases a disintegrin and metalloprotease with thrombospondin motifs-13 activity and reverses organ dysfunction in children with thrombocytopenia-associated multiple organ failure. Crit Care Med.

[52] Long EJ, Taylor A, Delzoppo C, Shann F, Pearson G, Buckley D (2013). A randomised controlled trial of plasma filtration in severe paediatric sepsis. Crit Care Resusc J Australas Acad Crit Care Med.

[53] Padmanabhan A, Connelly-Smith L, Aqui N, Balogun RA, Klingel R, Meyer E (2019). Guidelines on the use of therapeutic apheresis in clinical practice - evidence-based approach from the writing committee of the american society for apheresis: the eighth special issue. J Clin Apher.

[54] Mokrzycki MH, Kaplan AA (1994). Therapeutic plasma exchange: complications and management. Am J Kidney Dis.

[55] Shemin D, Briggs D, Greenan M (2007). Complications of therapeutic plasma exchange: a prospective study of 1727 procedures. J Clin Apher.

[56] Schmidt JJ, Asper F, Einecke G, Eden G, Hafer C, Kielstein JT (2018). Therapeutic plasma exchange in a tertiary care center: 185 patients undergoing 912 treatments - a one-year retrospective analysis. BMC Nephrol.

[57] Stahl K, Wendel-Garcia PD, Bode C, David S (2021). Unraveling the secret of re-balancing homeostasis in sepsis: a critical view on extracorporeal blood purification modalities. Intensive Care Med.

