# Supplementary material for: Clinical and biochemical endpoints and predictors of response to plasma exchange in septic shock: results from a randomized controlled trial
Source: Crit Care. 2022 May 12;26:134. doi: 10.1186/s13054-022-04003-2 (PMC9097091; doi:10.1186/s13054-022-04003-2)
Supplement: Supplementary file 1 — Additional file1: Table S1. Linear mixed effect model for the prediction of norepinephrine doses. Table S2. Linear mixed effect model for the prediction of serum lactate levels. Table S3. Linear mixed effect model for the prediction of the norepinephrine dose. [file 13054_2022_4003_MOESM1_ESM.docx]

**Supplementary Table 1: Linear mixed effect model for the prediction of norepinephrine doses**

|  | **Variance** | **Standard Deviation** | |
| --- | --- | --- | --- |
| **Random Effect** | | | |
| ***Intercept (Patient)*** | 0.05742 | 0.2396 | |
|  | **Estimate** | **Standard Error** | **p** |
| **Fixed Effects** | | | |
| ***Intercept*** | 0.676197 | 0.0694 | <0.0001 |
| **TPE (yes)** | -0.076538 | 0.097936 | 0.43769 |
| **Time (hours)** | -0.004517 | 0.003460 | 0.19440 |
| ***Interaction:* TPE (yes) - Time** | -0.013859 | 0.004730 | 0.00413 |

Abbreviations: TPE – Therapeutic Plasma Exchange

**Supplementary Table 2: Linear mixed effect model for the prediction of serum lactate levels**

|  | **Variance** | **Standard Deviation** | |
| --- | --- | --- | --- |
| **Random Effect** | | | |
| ***Intercept (Patient)*** | 8.643 | 2.940 | |
|  | **Estimate** | **Standard Error** | **p** |
| **Fixed Effects** | | | |
| ***Intercept*** | 5.07239 | 0.71572 | <0.0001 |
| **TPE (yes)** | -0.47049 | 1.01134 | 0.64410 |
| **Time (hours)** | 0.01234 | 0.02235 | 0.58198 |
| ***Interaction:* TPE (yes) - Time** | -0.09938 | 0.03047 | 0.00148 |

Abbreviations: TPE – Therapeutic Plasma Exchange

**Supplementary Table 3: Linear mixed effect model for the prediction of the norepinephrine dose.**

|  | **Variance** | **Standard Deviation** | |
| --- | --- | --- | --- |
| **Random Effect** | | | |
| ***Intercept (Patient)*** | 0.01141 | 0.1068 | |
|  | **Estimate** | **Standard Error** | **p** |
| **Fixed Effects** | | | |
| ***Intercept*** | 0.048532 | 0.111544 | 0.665 |
| **TPE (yes)** | 0.111065 | 0.167774 | 0.510 |
| **Time (hours)** | -0.010444 | 0.008373 | 0.215 |
| **Lactate (mmol/l)** | 0.014595 | 0.018802 | 0.434 |
| ***Interaction:* TPE (yes) – Time** | 0.008705 | 0.009162 | 0.344 |
| ***Interaction:* TPE (yes) - Lactate** | -0.010021 | 0.025420 | 0.512 |
| ***Interaction:* Time - Lactate** | 0.004819 | 0.001435 | 0.001 |
| ***Interaction:* TPE (no) – Norepinephrine (μg/kg/min)** | 0.797507 | 0.195952 | 0.0001 |
| ***Interaction:* TPE (yes) – Norepinephrine** | 0.585444 | 0.149183 | 0.0001 |
| ***Interaction:* Time – Norepinephrine** | -0.020622 | 0.017948 | 0.019 |
| ***Interaction:* TPE (yes) – Time – Lactate** | -0.005212 | 0.001773 | 0.004 |

Abbreviations: TPE – Therapeutic Plasma Exchange
